# FDA approves Icotyde^TM^ (Icotrokinra): a novel IL-23 receptor antagonist for plaque psoriasis

**DOI:** 10.1097/MS9.0000000000005234

**Published:** 2026-06-11

**Authors:** Muhammad Ali Abid, Muhammad Hafi Abid, Abdul Haseeb Hasan, Muhammad Daniyal Afzal

**Affiliations:** a Department of Medicine, King Edward Medical University, Lahore, Pakistan; b Department of Medicine, Faisalabad Medical University, Faisalabad, Pakistan; c Department of Medicine, Kyrgyz State Medical Academy, Bishkek, Kyrgyzstan

*Dear Editor,*

Plaque psoriasis is the most common form of psoriasis, presenting as erythematous plaques with silvery scales on the scalp and extensor surfaces of the body^[^1,2^]^. These plaques produce tiny pinpoint bleeding spots when removed^[^1^]^. The disease is characterized by periods of remission and exacerbation, typically triggered by trauma, infections, medications, or stress^[^2^]^. It is increasingly recognized not only as a dermatological condition but also as a systemic inflammatory disease involving multiple systems^[^1,2^]^. Other clinical variants of psoriasis include guttate psoriasis, erythrodermic psoriasis, inverse psoriasis, and pustular psoriasis, each with distinct clinical features and varying severity^[^1^]^. Psoriasis affects both males and females, with earlier onset seen in females and individuals with a positive family history^[^1^]^.

Psoriasis is driven by the interaction between dendritic cells, T-cells, and keratinocytes, with interleukin-23 (IL-23)/Th17 pathways acting as the key mediators for inflammation and rapid skin cell buildup^[^1,2^]^. Activated dendritic cells produce IL-23, which promotes the survival and differentiation of Th17 cells^[^1^]^. These cells secrete proinflammatory cytokines such as IL-22, IL-17F, and IL-17A, leading to keratinocyte hyperproliferation^[^1^]^. Tumor necrosis factor-alpha also plays a synergistic role in the amplification of inflammation^[^1,2^]^.

The cure for psoriasis has not been achieved, but several therapies are available for the management of skin involvement, including topical therapies, oral systemic therapies, phototherapy, and biologics^[^1^]^. Topical therapies remain the first line in the management of mild disease^[^1^]^. These include vitamin D analogs, corticosteroids, topical retinoids like tazarotene, and calcineurin inhibitors for sensitive areas^[^1^]^. These agents work by decreasing keratinocyte proliferation and reducing inflammation^[^1,2^]^. However, adherence can be limited due to local side effects or inconvenience^[^1,2^]^. For moderate disease, phototherapy (preferably narrowband ultraviolet B) along with conventional systemic agents, namely methotrexate, ciclosporin, and acitretin, are used^[^1^]^. Phototherapy induces T-cell apoptosis and alters the cytokine profile, while systemic agents act through varying immunomodulatory mechanisms^[^1^]^. Ciclosporin suppresses calcineurin-mediated T-cell activation, methotrexate inhibits dihydrofolate reductase and reduces T-cell activity, and acitretin modulates keratinocyte differentiation^[^1^]^. Despite their effectiveness, these therapies are limited by cumulative toxicity, organ-specific adverse effects, and a need for laboratory monitoring^[^1^]^.

The US Food and Drug Administration has recently approved Icotyde^TM^ (Icotrokinra) as an oral treatment for moderate-to-severe plaque psoriasis in patients ≥12 years who weigh at least 40 kg and are candidates for phototherapy or systemic therapy^[^3^]^. Icotrokinra is a novel, first-in-class IL-23 receptor antagonist that can be taken orally once daily to manage plaque psoriasis^[^3^]^. IL-23 is a key component of the immune signaling pathway involved in psoriasis-related inflammatory reactions^[^2^]^. By inhibiting this pathway, Icotrokinra helps reduce the inflammation that drives plaque formation and other symptoms of plaque psoriasis^[^2,3^]^.

The approval was based on Phase 3 ICONIC clinical development program, comprising four randomized controlled trials^[^3^]^. ICONIC-ADVANCE 1 (NCT06143878) and ICONIC-ADVANCE 2 (NCT06220604) enrolled 1505 adults in whom Icotrokinra was assessed against placebo for two co-primary endpoints: Investigator’s Global Assessment (IGA) 0/1 response and at least 90% improvement in the Psoriasis Area and Severity Index (PASI) from baseline (PASI 90)^[^4,5^]^. The results were evaluated at Week 16, where Icotrokinra showed improved outcomes compared to placebo^[^3–5^]^. ICONIC-LEAD (NCT06095115) and ICONIC-TOTAL (NCT06095102) included a total of 995 participants, out of whom 72 individuals were pediatric subjects aged ≥12 years who weighed at least 40 kg, proving its efficacy in the pediatric age-group as well^[^6,7^]^. The primary endpoints in ICONIC-LEAD (NCT06095115) were PASI 90 and IGA 0/1 response, while in ICONIC-TOTAL (NCT06095102), the primary endpoint was IGA 0/1 response^[^6,7^]^. Icotrokinra was evaluated against placebo and demonstrated improvements across all endpoints^[^6,7^]^. Icotrokinra is well-tolerated, with the most common adverse effects (≥1%) including headache, cough, fungal infections, fatigue, and nausea^[^3^]^. Treatment with Icotrokinra should be avoided in the presence of an ongoing clinically significant infection until the infection resolves or is adequately treated^[^3^]^. Potential adverse effects should be monitored in individuals with renal impairment and an estimated glomerular filtration rate <60 mL/min^[^3^]^.

Icotyde^TM^ (Icotrokinra) represents a newer therapeutic option for moderate-to-severe plaque psoriasis that may help address certain unmet needs, particularly in individuals with an inadequate response, intolerance, or contraindications to existing systemic and biologic therapies. By targeting key inflammatory pathways involved in psoriasis, it expands the available treatment landscape and offers an additional mechanism of action that may be useful in broadening therapeutic options. However, while clinical trial data demonstrate efficacy and adequate safety, long-term data is still required to better determine the durability of response, safety, and potential long-term adverse effects of the drug.

## Data Availability

Not applicable to this study.

## Research registration unique identifying number (UIN)

Not applicable to this study.
